# Pulmonary Effects of Adjusting Tidal Volume to Actual or Ideal Body Weight in Ventilated Obese Mice

**DOI:** 10.1038/s41598-018-24615-5

**Published:** 2018-04-24

**Authors:** Elise Guivarch, Guillaume Voiriot, Anahita Rouzé, Stéphane Kerbrat, Jeanne Tran Van Nhieu, Philippe Montravers, Bernard Maitre, Armand Mekontso Dessap, Mathieu Desmard, Jorge Boczkowski

**Affiliations:** 1 INSERM U955, Université Paris Est (UPEC), Faculté de Médecine, 94000 Créteil, France; 2 AP-HP, HU Hôpital Bichat-Claude Bernard, Département d’anesthésie-réanimation, 75018 Paris, France; 3 Hôpital Paris Saint Joseph, Service d’anesthésie, 75014 Paris, France; 4 AP-HP, HU Hôpital Tenon, Service de réanimation, 75020 Paris, France; 5 Université Paris Est Créteil (UPEC), Faculté de Médecine de Créteil, IMRB, GRC CARMAS, Créteil, 94000 France; 6 CHU Lille, Centre de Réanimation, Lille, 59000 France; 7 UPEC and AP-HP, HU Henri Mondor, Département de pathologie, 94000 Créteil, France; 8 AP-HP, HU Hôpital Henri Mondor, DHU A-TVB, Antenne de Pneumologie, 94000 Créteil, France; 9 AP-HP, HU Hôpital Henri Mondor, DHU A-TVB, Service de réanimation médicale, 94000 Créteil, France; 10 Centre hospitalier sud francilien, Service de réanimation, 91100 Corbeil-Essonnes, France; 11 INSERM UMR 1152, Faculté de médecine Paris Diderot Paris 7, 94000 Paris, France

## Abstract

Obese patients could be more susceptible to mechanical ventilation (MV)-induced lung injury than non-obese patients due to weight-dependent changes in lung properties. The aim of this study was therefore to evaluate the pulmonary effects of 2 hours low V_T_ MV in a diet-induced obese mice model, with V_T_ calculated on either the actual body weight (V_T_aw) or the ideal body weight (V_T_iw) . First, we hypothesized that a MV with V_T_aw would be associated with altered lung mechanics and an increased lung inflammation. Second, we hypothesised that a MV with a V_T_iw would preserve lung mechanics and limit lung inflammation. We analyzed lung mechanics and inflammation using bronchoalveolar lavage (BAL) cell counts, flow cytometry tissue analysis and histology. Lung mechanics and inflammation were comparable in control and obese mice receiving V_T_iw. By contrast, obese mice receiving V_T_aw had significantly more alterations in lung mechanics, BAL cellularity and lung influx of monocytes as compared to control mice. Their monocyte expression of Gr1 and CD62L was also increased. Alveolar neutrophil infiltration was significantly increased in all obese mice as compared to controls. In conclusion, our findings suggest that protective MV with a V_T_aw is deleterious, with a marked alteration in lung mechanics and associated lung inflammation as compared to lean mice. With V_T_iw, lung mechanics and inflammation were close to that of control mice, except for an increased alveolar infiltrate of polymorphonuclear neutrophils. This inflammation might be attenuated by a blunted recruitment of inflammatory cells associated with obesity.

## Introduction

Over the last several decades, the worldwide prevalence of obesity has steadily risen, reaching 36% in the United States in 2010^1^. Consequently, the number of obese inpatients requiring mechanical ventilation (MV), either in intensive care units or in the operating room, is increasing.

MV with excessive lung stress and strain may induce pulmonary adverse effects, called “ventilator-induced lung injury (VILI)”, due to barotrauma, volutrauma, atelectotrauma and biotrauma^2–4^, leading to increased mortality in patients with the acute respiratory distress syndrome (ARDS)^5^. Some evidence has suggested that high tidal volume (V_T_) may also be deleterious for patients with a healthy lung^6,7^, even for short periods of ventilation, as during general anesthesia^8–10^. Recent guidelines recommend limiting V_T_ to 6 to 8 mL/kg of ideal body weight (IBW) in patients with ARDS^11^, which tend to be extended to all critically ill patients^12^ and a V_T_ < 10 mL/kg of IBW has also been recommended during anesthesia^13,14^.

Despite the increasing number of ventilated obese patients, no specific study has been published concerning these characteristics of MV. Guidelines advocate the use of the IBW to calculate the V_T_, but the formulas are unfamiliar to medical doctors and are not fully integrated into current practice. Indeed, it has been recently demonstrated that obesity is a risk factor for being ventilated with a large V_T_ (>10 mL/kg of IBW)^15,16^, with an increased risk of secondary ARDS in obese ICU patients^17^.

Moreover, obese patients might be more susceptible to VILI, even when ventilated with a low V_T_, because of both mechanical and biological characteristics of this condition. Firstly, obese individuals have a lower thoraco-pulmonary compliance because of fatty infiltration of the chest, which induces increased airway pressures, and their lungs may be not uniformly inflated during MV because of greater basal atelectasis^18^. Secondly, obesity is associated with a baseline low-grade inflammatory status, which might increase vulnerability to biotrauma^19–21^. Thus, low serum adiponectin levels have been shown to promote acute lung injury in obese mice^22^. It can therefore be hypothesized that obese subjects may be more prone to MV-associated lung injuries than non-obese subjects, with low V_T_ ventilation calculated on actual weight or even calculated on IBW. However, to the best of our knowledge, this hypothesis has not been previously investigated despite the important implications in terms of public health given the continuously growing prevalence of obesity.

The aim of this study was therefore to evaluate the pulmonary effects of low V_T_ MV in a diet-induced obese mice model, with V_T_ calculated on either the actual body weight or the ideal body weight. First, we hypothesized that a MV with a V_T_ calculated on the actual body weight would be associated with altered lung mechanics and an increased lung inflammation. Second, we hypothesized that a MV with a V_T_ calculated on the ideal body weight would preserve lung mechanics and limit lung inflammation as compared to a V_T_ calculated on the actual body weight. Some of our results have been previously reported in abstract form^23^.

## Results

### Diet-induced obesity model

After the 12-week feeding period, the weight and BMI of all mice fed a high-fat diet (non-ventilated obese (NVO) + obese mice ventilated with V_T_ based on actual weight (OV_T_aw) + obese mice ventilated with V_T_ based on ideal weight (OV_T_iw)) was higher than that of mice fed a low-fat diet (non-ventilated controls (NVC) + ventilated controls (VC)): 41.1 [38.6–45.9]g *vs* 30.9 [29.5–32;1]g, *p* < 0.0001. Their body mass index (BMI) was also increased (0.40 [0.38–0.44] *vs* 0.31[0.30–0.32] g/cm^2^, *p* < 0.0001) (Table 1).

**Table 1:** Weight characteristics of the mice in the various groups.

	**NVC** (n = 22)	**VC** (n = 24)	**NVO** (n = 21)	**OV**_**T**_**aw** (n = 19)	**OV**_**T**_**iw** (n = 21)
Weight (g)	31.0 [29.5–32.1]	30.8 [29.2–32.2]	40.8 [37.7–42.6]	42.0 [39.3–48.3]	41.2 [38.6–45.3]
BMI (g/cm^2^)	0.31 [0.30–0.33]	0.31 [0.30–0.32]	0.39 [0.37–0.41]	0.43 [0.39–0.46]	0.41 [0.38–0.44]

Mann-Whitney test and Kruskal-Wallis test were not significant between the control groups and between the obese groups, respectively. BMI: Body Mass Index.

### Baseline characteristics of obese and control mice

**Figure 1:**
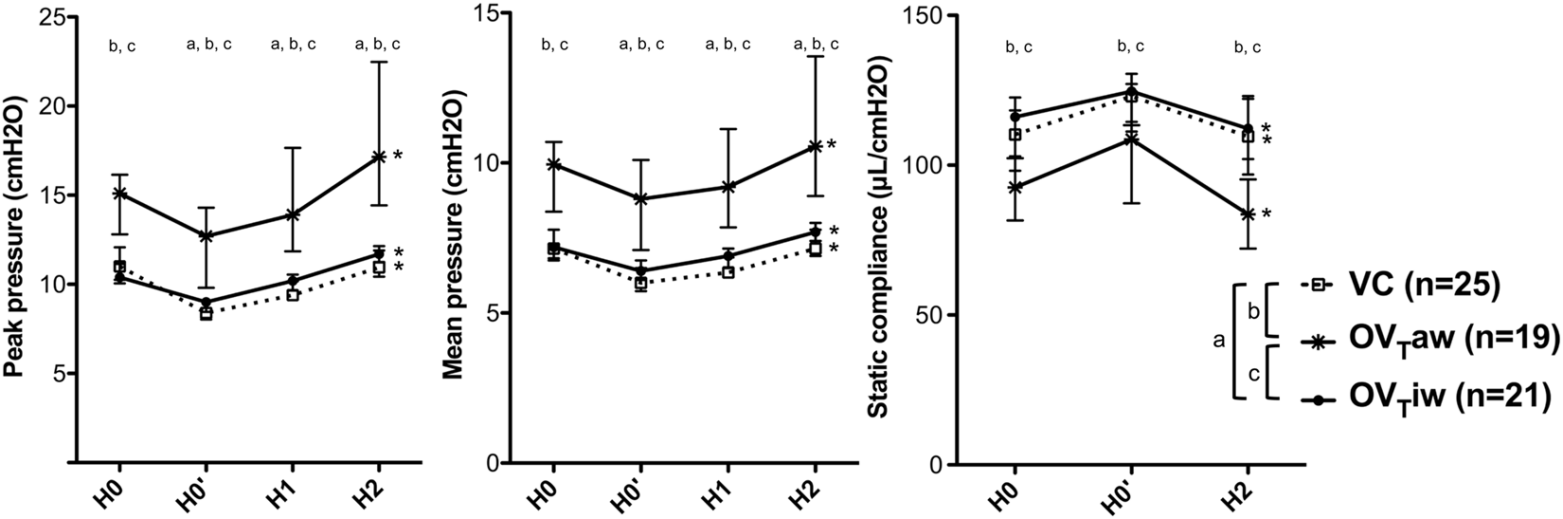
Variations of peak pressure, mean pressure and static compliance during the two hours of ventilation. H0: On connection of the ventilator, H0’: after recruitment maneuver, H1: after one hour of ventilation, H2: after 2 hours of ventilation. a, b and c denote a *p* value (Mann-Withney pairwise comparisons after Kruskal-Wallis test) <0.05 for the following pairwise comparisons: for VC *vs* OV_T_iw, VC *vs* OV_T_aw and OV_T_aw *vs* OV_T_iw, respectively. *Denotes *p* value (Mann-Withney pairwise comparison) <0.05 between H2 and H0’ in each group (n = 19–25 per group).

Pulmonary inflammation, assessed by BAL cell counts, histologic score and inflammatory cells in lung tissue quantified by flow cytometry, did not differ between NVO and NVC mice (Table 2). Alveolar permeability, assessed by the BAL protein concentration, was also similar in the two groups (196 [183–228] in NVO mice *vs* 198 [188–226] µg/mL in NVC mice, *p* = 0.84). Lung mechanics measured at the beginning of the ventilation period showed that obese (OV_T_iw) and control (VC group) mice displayed similar compliance (125 [115–131] *vs* 123 [111–127] µL/cmH_2_O, *p* = 0.49). After recruitment maneuvers, obese mive (OV_T_iw and OV_T_aw) had higher peak and mean airway pressures than VC mice and OV_T_aw mice had higher peak and mean airway pressures than OV_T_iw mice (Fig. 1).

**Table 2:** Characteristics of pulmonary inflammation in control and obese mice before ventilation.

	NVC (n = 9–11)	NVO (n = 9–11)	*p*
BAL cell count (cells/mL)	80,000 [68,300–95,556]	73,333 [64,444–88,889]	0.60
BAL neutrophil (cells/mL)	444 [0–683]	0 [0–433]	0.16
Macrophages (10^3^ cells/lungs)	519 [394–723]	560 [499–1018]	0.11
Neutrophils (10^3^ cells/lungs)	882 [453–2,083]	1,374 [912–2,555]	0.36
Monocytes (10^3^ cells/lungs)	246 [115–636]	375 [230–534]	0.34
Histologic inflammation score	3.0 [2.5–7.5]	9.0 [6.0–11.0]	0.08

Pulmonary inflammation was characterized by BAL cell counts, number of inflammatory cells counted by flow cytometry and a histologic inflammation score. BAL: Bronchoalveolar lavage.

### Lung mechanics during MV

Because the mean weight of VC mice was used to calculate the V_T_ applied in OV_T_iw mice, the two groups had an identical V_T_ of 0.241 [0.230–0.252] mL, which was significantly lower than the V_T_ received by OV_T_aw mice (0.331 [0.304–0.376]mL, *p* < 0.0001). Lung mechanics slightly deteriorated during the 2-hour MV period in the 3 ventilated groups, with a increase in airway pressures and a decrease in respiratory system compliance (Fig. 1). Deterioration of lung mechanics during the MV period was more pronounced in the OV_T_aw group, with a ΔH2-H0’ higher for peak and mean airway pressures (*p* = 0.00015 *vs* VC and OV_T_iw) and lower for the respiratory system compliance (*p* = 0.04 *vs* VC and *p* = 0.02 *vs* OV_T_iw after corrections for multiple testing).

### BAL fluid cell counts and protein concentration

**Figure 2:**
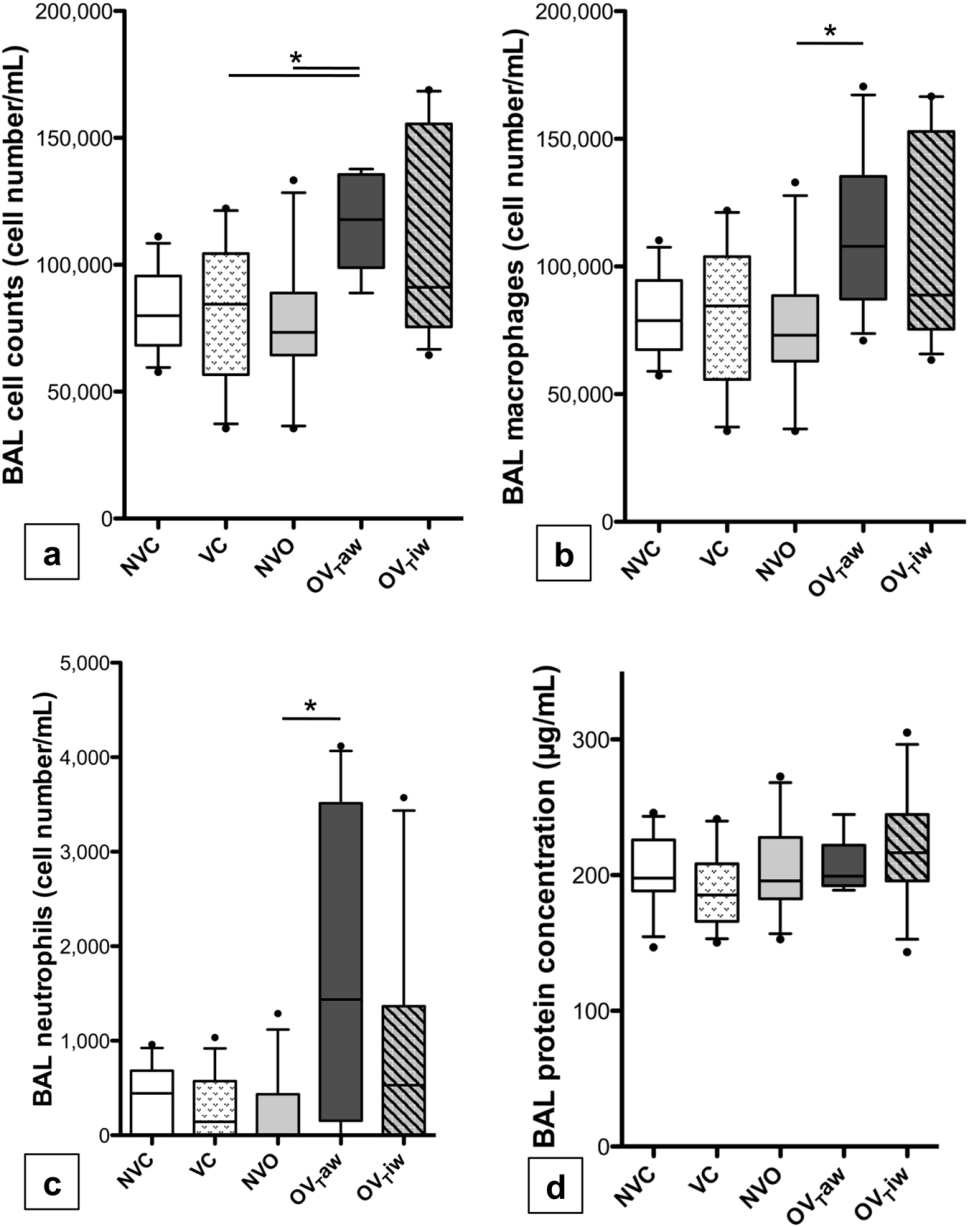
(**a**) Bronchoalveolar lavage (BAL) cell counts in the 5 groups of mice. (**b**,**c**) Number of macrophages (**b**) and neutrophils (**c**) in BAL. (**d**) BAL protein concentration. *p < 0.05 between the 2 groups after correction for multiple comparisons (n = 13–14 per group).

Inflammatory cells in BAL fluid were almost exclusively macrophages (Fig. 2). The global cell count and the differential cell count of macrophages did not differ between VC and OV_T_iw groups. There was a a higher global cell count in the OV_T_aw group as compared to the VC and NVO groups (*p* = 0.0287 and 0.0238 after correction for multiple testing, respectively). These cells were macrophages and neutrophils, as macrophages and neutrophils cell counts were higher in the OV_T_aw group as compared to the NVO group (*p* = 0.0455 and 0.0434, respectively) and tend to be higher as compared to the VC group (*p* = 0.0959 and 0.0602 after correction for multiple testing, respectively). The BAL protein concentration did not differ between the three groups (Fig. 2).

### Inflammatory cell subpopulations and their activation by flow cytometry in lung tissue

**Figure 3:**
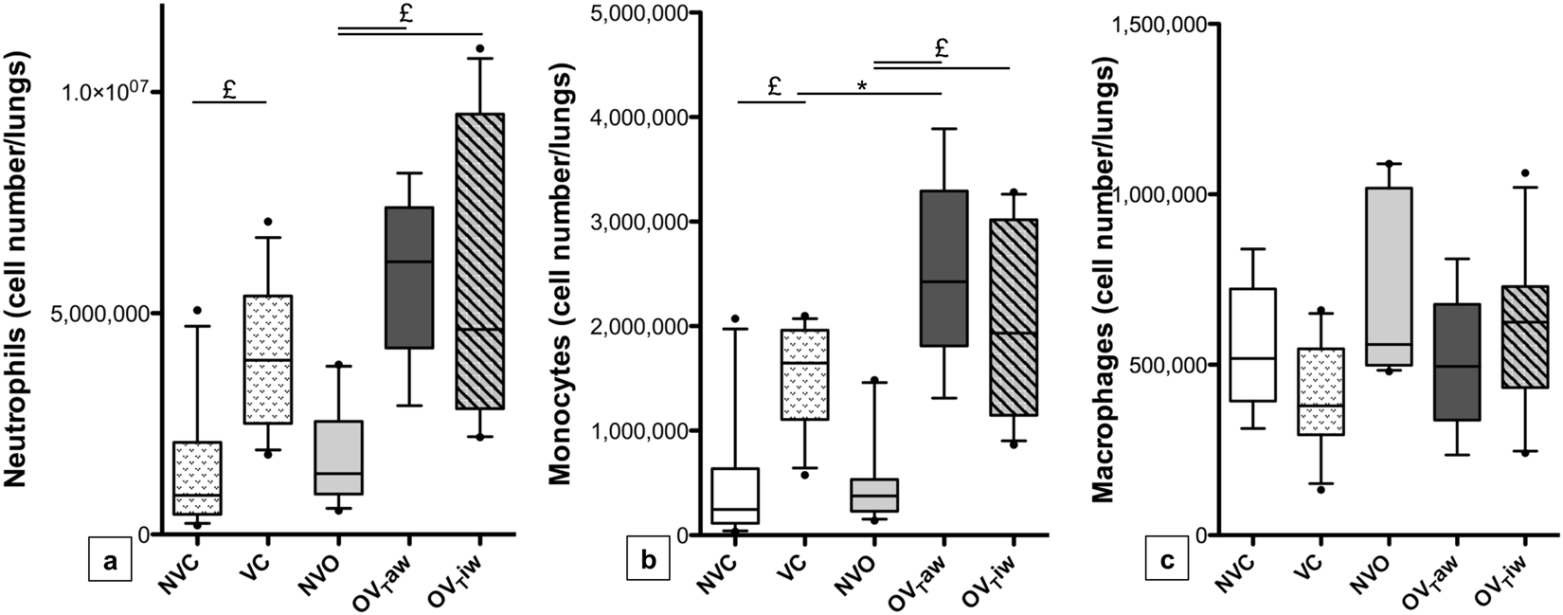
Numbers of neutrophils (**a**), monocytes (**b**) and macrophages (**c**) observed by flow cytometry in lung tissue in the 5 groups of mice. *p < 0.05; ^£^*p* < 0.01 (n = 8–13 per group).

Both obese and control mice exposed to MV displayed higher monocyte and neutrophil counts in lung tissue, compared to non-ventilated mice (VC *vs* NVC, OV_T_aw *vs* NVO and OV_T_iw *vs* NVO comparisons). Similar macrophage counts were observed in the ventilated and non-ventilated groups. Within ventilated mice, we observed a higher monocyte count in lung tissue of OV_T_aw group (but not OV_T_iw animals) as compared to the VC group. Macrophage and neutrophil counts in lung tissue were not significantly different between the three ventilated groups (OV_T_aw, OV_T_iw and VC groups), but neutrophils also tend to be higher in OV_T_aw as compared to VC (*p* = 0,0602 after correction for multiple testing) (Fig. 3).

**Figure 4:**
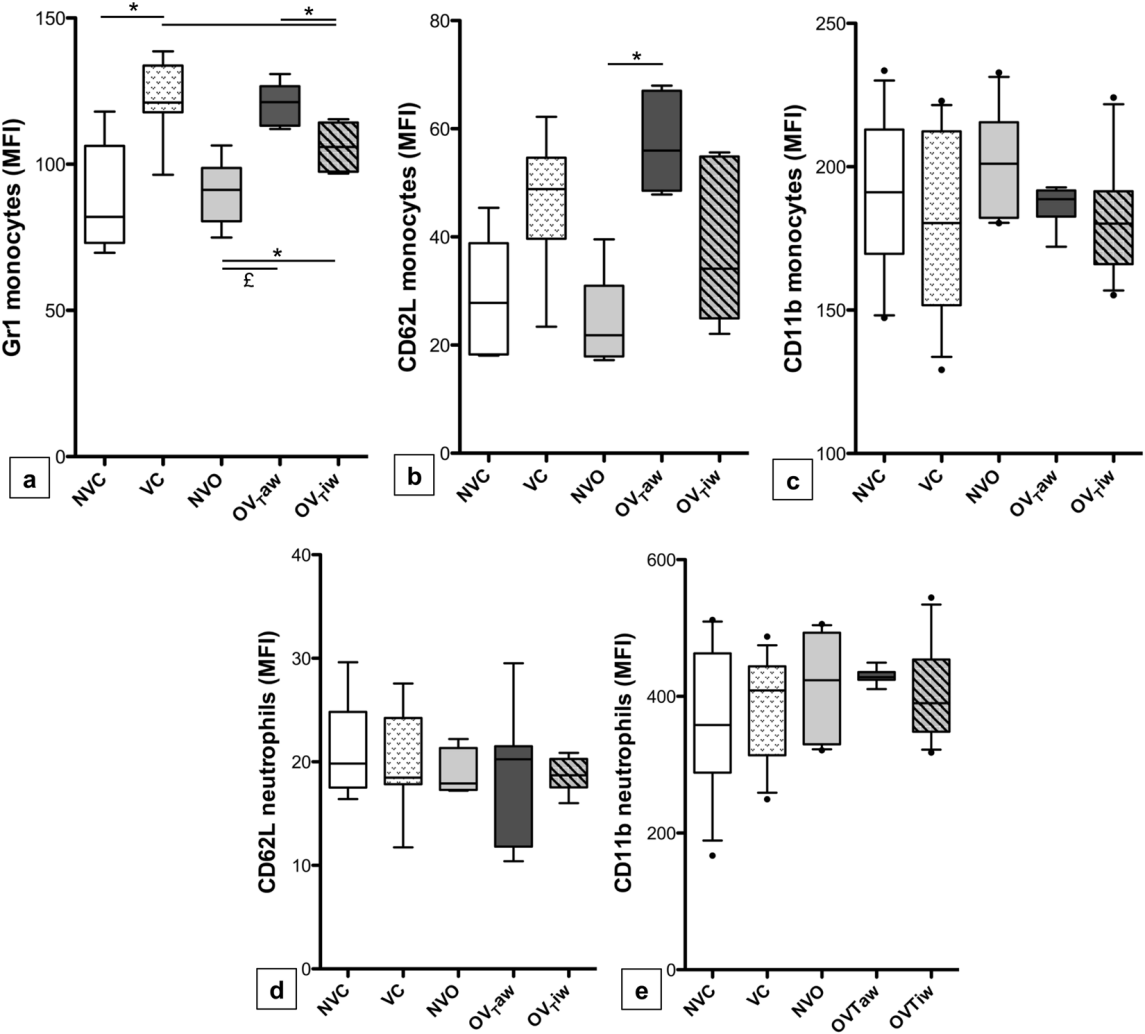
Gr-1 (**a**), CD62L (**b**) and CD11b (**c**) expression on pulmonary monocytes and CD62L (**d**) and CD11b (**e**) expression on pulmonary neutrophils expressed as mean fluorescent intensity (MFI). *p < 0.05, ^£^*p* < 0.01 (n = 8–13 per group).

Concerning the degree of activation of the inflammatory cells, no difference in neutrophil CD11b and CD62L expression was observed between the 3 ventilated groups (Fig. 4d,e). In contrast, high monocyte activation was observed in OV_T_aw mice, as the expression of Gr-1 on monocytes was higher in the OV_T_aw group than in both the NVO and OV_T_iw groups. Moreover, the expression of CD62L on monocytes was higher in the OV_T_aw group than in the NVO group, whereas no difference was observed between the VC and NVC groups on one hand and between the OV_T_iw and NVO groups on the other hand (Fig. 4a,b). Interestingly, Gr-1 expression on monocytes was less intense in OV_T_iw mice than in VC mice (Fig. 4a).

### Quantification of lung tissue neutrophil infiltration

**Figure 5:**
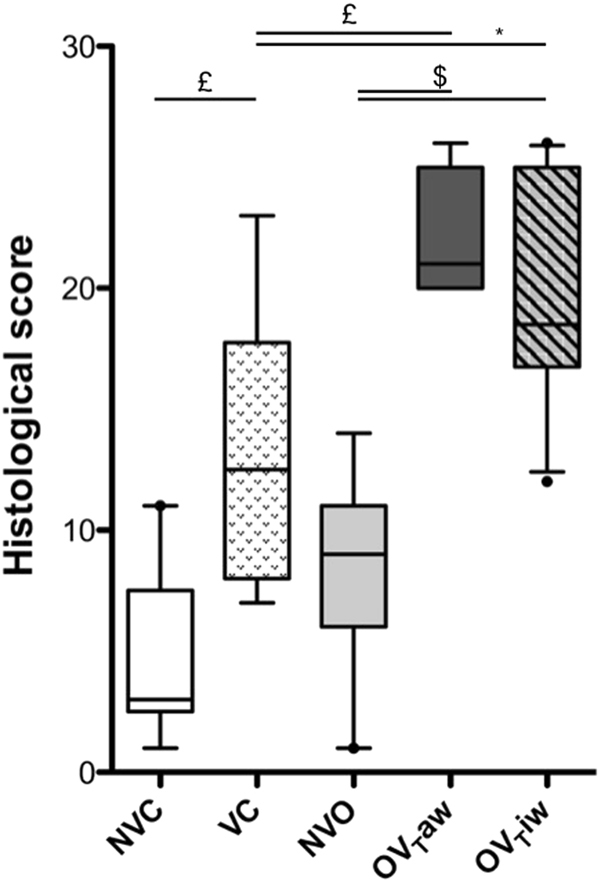
Alveolar neutrophil infiltration represented by a histologic score in the 5 groups (n = 7–10 per group). *p < 0.05; ^£^*p* < 0.01; ^$^*p* < 0.001.

**Figure 6:**
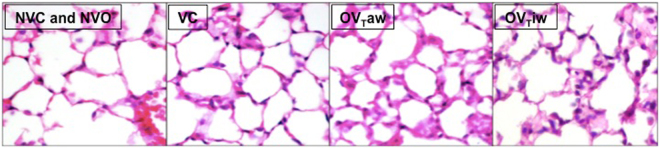
Representative 5 µm sections of alveolar neutrophil infiltration. Hematoxylin-eosin staining, 40-fold magnification NVC or NVO: low or no infiltration, VC: moderate infiltration, OV_T_aw and OV_T_iw: significant neutrophil infiltration.

Ventilated mice displayed higher neutrophil infiltration in the lungs compared to non-ventilated mice (VC *vs* NVC, OV_T_aw *vs* NVO and OV_T_iw *vs* NVO) (Fig. 5). This lung injury was particularly marked in obese mice, as the histologic score was higher in both the OV_T_aw and OV_T_iw groups compared to the VC group. No significant difference was observed between the OV_T_aw and OV_T_iw groups. Figure 5 represents the values of the histologic score and Fig. 6 shows representative sections of alveolar neutrophil infiltration.

## Discussion

This study shows that ventilating obese mice with a V_T_ calculated on their actual weight is deleterious, with a broader alteration of ventilatory mechanics, BAL total cell count, monocyte cell count and activation in lung tissue.

When V_T_ was calculated using the actual weight, ventilatory mechanics were considerably impaired compared to ventilated controls, achieving the highest airway pressures and lowest compliance at H2. This cannot be explained *a priori* by more atelectasis, as these obese mice were subjected to the same level of PEEP. The most likely hypothesis is the presence of interstitial and alveolar edema, thereby inducing alveolar rigidity and decreased compliance, which may correspond to an early manifestation of ventilator-induced lung injury^24^ in OV_T_aw mice. Although we did not demonstrate increased alveolar-capillary permeability in this group, it displayed increased BAL total cell count. Analysis on flow cytometry revealed an increase in recruited monocytes as compared to VC, and the number of neutrophils also tends to be higher. Moreover, monocytes of OV_T_aw displayed more intense L-selectin expression as compared to their non-ventilated counterparts, whereas no difference was observed between VC and OV_T_iw mice on one hand and VC and NVC on the other hand. Wilson *et al*.^25^ have previously shown that aggressive ventilation (35 mL/kg of V_T_ in their model) and, to a lesser extent, protective ventilation, induced an influx of immature Gr-1^high^ monocytes from the bone marrow. These monocytes also overexpressed L-selectin, allowing them to migrate out of the bloodstream into the lung. Similar results were observed in our study, highlighting the harmful effect of a supposed “protective” V_T_ calculated using the actual weight of obese mice. On histologic examination, we also demonstrated increased alveolar inflammation in OV_T_aw mice, with a higher neutrophil infiltration score compared to VC mice. The lack of significant increase in neutrophil by cytometry could be due to the short duration of the experiment (2 hours) that dit not allow monocytes time to differentiate into neutrophils and/or the lack of infectious challenge.

Obese mice ventilated with a V_T_ calculated using the ideal body weight had lung mechanics and inflammation parameters close to control mice except for a higher alveolar infiltration of neutrophils on histological score, which was similar to OV_T_aw, and a lower Gr-1 expression on monocyte. The decrease in most inflammatory parameters in the OV_T_iw group as compared to OV_T_aw may be ascribed to the reduction in V_T_ with the use of ideal weight, but the higher neutrophilic alveolar infiltration on histological score do not corroborate this hypothesis. An alternative hypothesis, supported by some publications, could be a role of obese phenotype in this decreased monocyte activation, as obese individuals appear to present a degree of immune deficiency, with delayed secretion of cytokines and recruitment of inflammatory cells in response to stimulation by a pathogen^26,27^, which could explain decreased activation of monocytes in OV_T_iw mice compared to VC mice, while alveolar infiltration by inflammatory cells was increased. Recent reports have highlighted abnormal diapedesis in obese mice exposed to inhalation of LPS^28^ or ovalbumin^29^ with increased influx of neutrophils and eosinophils in blood and lung tissue, but decreased BAL cell counts and cytokines. This phenomenon could also explain our finding of excess neutrophils observed in the alveolar walls, but not in the BAL of OV_T_iw mice. Overall, our findings may suggest that protective ventilation in obese subjects, even when V_T_ is calculated using the IBW, induces an increased lung inflammation as compared to that observed in lean subjects, but this inflammation could be attenuated by a blunted recruitment of inflammatory cells associated with obesity.

This study presents several limitations. First, we used a short (2 hours) MV. However, a short period (2 to 3 hours) of low V_T_ MV was shown to cause lung inflammation and edema in uninjured mice^30^, whereas a short period of high V_T_ MV was shown to provide much more lung injury^31–33^. Second, we used a minimal PEEP (1.5 cmH_2_O). This might be critized since numerous investigations encourage to use higher PEEP in the operating room, especially in obese patients^34^. However, other teams applied a similar low PEEP (below 2 cmH_2_O) during short MV in mice^32,35,36^. Here, with a PEEP settled at 1.5 cmH2O, we observed a slight deterioration of lung mechanics over the MV period. Indeed, the static compliance after 2 hours of MV was closed to that one recorded at time of connexion to the ventilator in both the VC and OV_T_iw groups. In another work by our group, we showed that the dynamic compliance decreased below the H0 value after 3 hours of mechanical ventilation in uninjured mice^37^. Therefore, a 1.5 cmH_2_O PEEP looked suitable in such a low-V_T_ short MV applied to uninjured mice. Third, our model of obesity was induced by diet, with a moderate weight gain, as compared to some genetic models^38^. This moderate weight gain may have precluded hightlighting meaningful differences between obese and control groups. However, this model is much closer to clinical reality in humans and genetic models like *ob/ob* and *db/db* type could not be used in this study, as these animals present early weight gain that induces an abnormal development of the lungs, leading to a twofold lower lung volume than in control mice^39^. This discrepancy would have induced a major bias in the interpretation of the ventilatory mechanics. Fourth, we used the mean weight of control mice to calculate the ideal weight of obese mice. However, no formula is available to calculate IBW in mice. In addition, the naso-anal length of obese and control mice was similar in our study (*data not shown*). Fifth, some of our results (BAL cell count and flow cytometry neutrophils comparisons) were no longer statistically significant after correction for multiple testing, due to the low number of animals and the number of study groups. However, the corrected *p* values were “marginally significant”; overall, our results suggested that, with a short-term ventilation, the conjunction of obesity and a V_T_ calculated on the actual weight induce a significant alveolar inflammation. Finally, we did not use an infectious challenge like lipopolysaccharide, that could mimic the clinical scenario in intensive care where pneumonia is a major cause of mechanical ventilation. This lack of infectious challenge could in part explain the absence of enhanced inflammatory state in diet-induced obese mice at baseline before starting mechanical ventilation, like previously described in this model^40^. However, other studies have demonstrated the absence of overt lung inflammation in unchallenged obese mice^41,42^. Further experimental studies are needed to assess the exact impact of mechanical ventilation using the actual *vs* ideal body weight in terms of recruitment and migration of inflammatory cells (which could be blunted in obese individuals) and when using an infectious challenge like lipopolysaccharide.

Our study has some clinical implications. These data suggest systematically calculating the IWB of obese patients, both in intensive care and in the operative room, even for short periods of ventilation. However, further studies are required to assess the exact impact of protective mechanical ventilation on the IBW of obese subjects, as it appears to induce more marked pulmonary inflammation compared to controls, but the presence of secondary lesions must be determined. A deficiency in the recruitment and migration of inflammatory cells in obese subjects could also play a protective role against lung injury induced by inflammation secondary to mechanical ventilation. At present, clinical studies investigating the morbidity and mortality of ventilated obese subjects, often retrospective and comprising numerous biases, especially disparities between the management of obese and non-obese patients, have reported contradictory results^5,43–46^. Acute lung injury or ARDS may also have been over-diagnosed due to poor quality radiographs or undiagnosed atelectasis.

In conclusion, we herein demonstrate, for the first time, the harmful effect of a two hours ventilation strategy with tidal volume of 8 mL/kg calculated on the actual weight in obese mice. When the tidal volume was calculated using the ideal weight, lung mechanics and inflammation were close to that of control mice, except for an increased alveolar infiltrate of neutrophils on histology. Further studies are needed to determine the effect of protective ventilation based on IBW on the morbidity and mortality of these patients.

## Material and Methods

Additional details on the methods are provided online (see supplementary information file, SIF).

### Animal protocol

Five-week-old male C57/Bl6 mice were purchased from the Janvier laboratory (Le Genest-Saint-Isle, France) and were housed in our “small animal” facility. They received one week of adaptation with their usual food. At the age of 6 weeks, they were divided into two groups. One group (control group) received a diet in which 10% of the calories were derived from fat in the form of lard (D12450B, SSNIFF, Soest, Germany), while the other group (obese group) was fed a diet in which 60% of the calories were derived from fat in the from of lard (D12492, SSNIFF). Both groups were given food and water *ad libitum* and were exposed to a 12:12 h light-dark cycle. At the age of 18 weeks and after 12 weeks of the diet, animals were weighed and then fasted for 12 hours with free access to water before the experimentation. The body mass index (BMI) was calculated as previously described formula: BMI = weight (g)/(naso-anal length (cm))^2 ^^47^. Mice were then anesthetized with intraperitoneal pentobarbital (30 µg/g of body weight, Hospira, Meudon La Forêt, France) followed by continuous 1.5% isoflurane (Abbott, Rungis, France). The larynx was surgically exposed and the trachea was intubated orally under direct vision with a metal cannula (inner diameter of 1 mm, Harvard Apparatus, Les Ulis, France). The tracheal cannula was properly secured with surgical thread (Ethicon 3–0, Ethicon, Auneau, France) before connection to mechanical ventilator in order to avoid leaks.

### Mechanical ventilation

After intubation, animals were left for 5 min in spontaneous breathing and half of the mice (obese and control) were sacrificed. The other half of were ventilated by means of a small-animal ventilator (flexiVent, Scireq, Montreal, Canada) as follows: V_T_ = 8 mL/kg of body weight, respiratory rate = 180/min, end-expiratory pressure = 1.5 cmH_2_O, and inspired fraction of oxygen = 0.4–0.6. This combination of V_T_ (8 ml/kg) and respiratory rate (180/min) has been shown to produce adequate minute ventilation and PaCO_2_ in mice^48^. Mechanical ventilation lasted 2 hours with continuous anesthesia maintained by 1.5% isoflurane and muscle paralysis using intraperitoneal pancuronium at the onset of the experiment (0.8 mg/kg of body weight, Organon, Puteaux, France) to ensure passive mechanical conditions. Body temperature was monitored using a rectal probe and was maintained at 36.5 °C with a blanket connected to a homeothermic regulator (Homeo-blanket system 50–7221 F, Harvard Apparatus, Les Ulis, France). Mice received one intraperitoneal warm fluid bolus (physiological serum) at the onset of the experiment (20 mL/kg of body weight). The dosage of all medications administered was calculated on actual weight. In ventilated obese mice, tidal volume was calculated either on actual weight or ideal weight. As no formula is available to calculate predicted body weight in mice, ideal weight was based on the mean weight of control mice (namely 31.2 g) (see SIF for details).

### Experimental design

**Figure 7:**
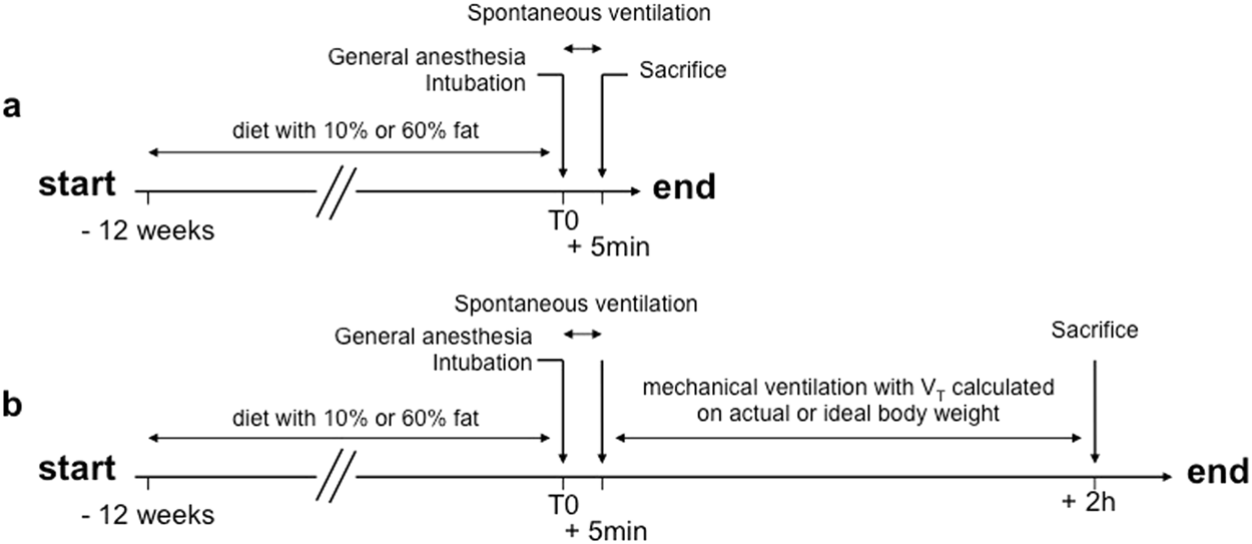
Design of experimental groups. (**a**) Mice were fed either a diet containing 10% fat (control mice) or 60% fat (obese mice) for 12 weeks. Non-ventilated control or obese mice (NVC and NVO) underwent general anesthesia, were intubated and were sacrificed after 5 min of spontaneous ventilation. (**b**) Mice were fed the same diet (10% or 60% fat). Ventilated control and obese mice underwent general anesthesia, were intubated and were sacrificed after 2 hours of mechanical ventilation, with a tidal volume calculated on actual weight for control mice (VC) or on either actual weight (OV_T_aw) or ideal weight (OV_T_iw) for obese mice.

The experimental design included five groups: NVC (non-ventilated control mice), VC (ventilated control mice), NVO (non-ventilated obese mice), OV_T_aw (ventilated obese mice with V_T_ calculated on the actual weight) and OV_T_iw (ventilated obese mice with V_T_ calculated on an ideal weight) (Fig. 7). The number of animals used in each experiment is detailed in the legend of all result table or figure.

### Respiratory mechanics

Mean and peak airway pressures were recorded at initiation of mechanical ventilation and then hourly throughout the experimental period. Quasi-static compliance of the respiratory system was determined from a continuous pressure-volume curve performed at initiation of mechanical ventilation and at the end of MV.

### Specimen collection

After 2 hours of MV, mice were sacrificed by exsanguination. Two different specimen collection procedures were used: For one half of the mice, immediately following thoracotomy, the pulmonary circulation was flushed by injection of 2 mL of saline into the right ventricle, and the lungs were then inflated by tracheal injection of 4% formaldehyde at a pressure of 20 cmH_2_O, fixed in 4% formaldehyde and finally paraffin-embedded. For the other half of the mice, bronchoalveolar lavage was performed with two tracheal injections of 1 mL of saline via the tracheal tube. A pulmonary circulation flush was then performed as previously described and both lungs were mechanically disrupted for flow cytometry analysis.

### Histologic examination

After formalin fixation and paraffin embedding, 5 µm thick sections of the left lung were cut and stained with hematoxylin, eosin and saffron (HES). An optical microscope (BX51 Olympus) with camera (Camedia 5060, Olympus) was used for histologic examination and photographs. An inflammatory score, derived from the VILI score^49,50^, assessing the degree of neutrophil infiltration in alveolar spaces, was determined on each lung (see SIF for details). In practice, HES-stained lung sections were examined at high power field (x40) and only alveolar spaces, located at a distance of more than one alveolus from bronchial spaces, were analyzed. Ten random fields were examined and the neutrophil count was scored from 0 to 3: 0 for <5 neutrophils per field, 1 for 5 to 10 neutrophils per field, 2 for 10 to 20 neutrophils per field, and 3 for >20 neutrophils per field. All lung sections were examined under blinded conditions, without knowledge of the mouse status (obese or not, ventilated or not).

### Bronchoalveolar lavage

The total cell count was determined for a fresh fluid specimen using slides with counting grids (Hycor biomedical, Indianapolis, IN, USA). The cell pellet was diluted in saline, and differential cell counts were performed on cytospin preparations (Cytospin 3; Shandon Scientific, Cheshire, UK) stained with Diff-Quick stain (Baxter Diagnostics, McGaw Park, IL, USA). Bronchoalveolar lavage fluid was centrifuged (1500 rpm, 13 min at 4 °C), and cell-free supernatants were stored at −80 °C for subsequent assessment of protein content (using a colorimetric BCA assay, Thermo Scientific, Rockford, IL, USA).

### Flow cytometric analysis

After performing BAL, a pulmonary circulation flush was performed as previously described and both lungs were removed and placed in 2 mL of cell culture medium (RPMI 16/40 supplemented with 0.1% L-glutamine and 0.1% of nonessential amino acids) and then in ice. At the end of the experiment, lungs were placed in the same medium with 5% fetal bovine serum (FBS), 0.35 mg/mL collagenase XI (Sigma-Aldrich, Saint Louis, MO, USA) and 5 mg/mL type IV bovine pancreatic DNAse (Sigma-Aldrich), mechanically disrupted with a scalpel, and placed in an incubator at 37 °C with 5% CO_2_ for 35 min. The action of the enzymes was stopped by adding 9 mL of RPMI medium containing 10% FBS, and digested lungs were further disrupted by gently pushing the tissue through a nylon screen (70 µm). The single-cell suspension was then washed and centrifuged at 900 rpm. To lyze contaminating red blood cells, the cell pellet was incubated for 5 min at room temperature with 5 mL of Gey’s solution (NH_4_Cl and KHCO_3_). Cells were then washed with PBS, recentrifuged and the pellet was resuspended in 4 mL of PBS. Cell count was then performed using slides with counting grids (Hycor biomedical) after Trypan blue staining.

In cytometry tubes, 500,000 cells in 50 µL were incubated with 50 µL of Fc Block (Anti-CD16/CD32 antibody, BD Biosciences, San Jose, CA, USA) at 4 °C for 15 min and then stained with fluorophore-conjugated anti-mouse antibodies for CD11b, CD11c, Gr-1 (Ly6C/G), F4/80 and CD62L (L-selectin) or appropriate isotype-matched controls.

Samples were analyzed using a CyAn cytometer and Summit software (Beckman Coulter, Brea, USA). Macrophages were identified as high CD11c and low CD11b cells. In order to differentiate and quantify monocytes, low side-scatter, high CD11b and low CD11c events were gated. Neutrophils were differentiated from monocytes as high side-scatter, high CD11b and low CD11c cells. F4/80 was used to facilitate differentiation between monocytes and neutrophils. Cell activation was assessed based on L-selectin and CD11b adhesion molecule expression for neutrophils and L-selectin, CD11b and Gr-1 expression for monocytes. Details of the leukocyte identification procedure are represented in Supplementary Fig. S1.

### Statistical analysis

Data were analyzed using Prism software version 5.0 (San Diego, CA, USA). Continuous data were expressed as median [IQ25-IQ75]. Statistical comparisons between two groups used the Mann-Whitney or the Wilcoxon test. Statistical comparisons between more than two groups used the Kruskal-Wallis test followed by a pairwise Mann-Whitney test with Benjamini-Hochberg correction for multiple testing. Two-tailed *p* values less than 0.05 were considered significant.

### Ethical approval

All the experiments were performed in accordance with the official regulations of the French Ministry of Agriculture and the US National Institute of Health guidelines for the experimental use of animals, were approved by the animal institutional review board of the Paris-Est Creteil Val de Marne University, and were conducted in a specific “little animal” plateform (Plateforme Exploration Fonctionnelle du Petit Animal, INSERM U955, Créteil, France).

## Electronic supplementary material

Supplementary information
