# Supplementary material for: Pulmonary Effects of Adjusting Tidal Volume to Actual or Ideal Body Weight in Ventilated Obese Mice
Source: Sci Rep. 2018 Apr 24;8:6439. doi: 10.1038/s41598-018-24615-5 (PMC5915403; doi:10.1038/s41598-018-24615-5)
Supplement: Supplementary file 1 — Supplementary information [file 41598_2018_24615_MOESM1_ESM.pdf]

SUPPLEMENTARY INFORMATION

Pulmonary Effects of Adjusting Tidal Volume to Actual or Ideal Body Weight in Ventilated Obese Mice

Elise Guivarch, Guillaume Voiriot, Anahita Rouzé, Stéphane Kerbrat, Jeanne Tran Van NHieu, Philippe Montravers, Bernard Maitre, Armand Mekontso Dessap, Mathieu Desmard, Jorge Boczkowski

Mechanical ventilation

Mice were ventilated in the supine position using humidified gas (20 mg H_2O/L absolute humidity, MR410 humidifier, Fischer & Paykel Healthcare, Courtaboeuf, France), with a tidal volume of 8 mL/kg of body weight, a respiratory rate of 180/min, 1.5 cmH_2O end-expiratory pressure, and FiO_2 of 0.4-0.6, by means of a computer-driven small-animal ventilator (flexiVent, Scireq, Montreal, Canada).

Respiratory mechanics

Special features of the FlexiVent© ventilator include continuous monitoring of airway pressures and a precision computer-controlled piston that is capable of accurately measuring the delivered volume (with appropriate corrections for gas compression) and to produce any desired waveform, allowing the assessment of respiratory mechanics by the forced oscillation technique (FOT) and pressure-volume curves [1]. Before connecting each mouse to the ventilator, pressure and piston displacement calibration data were collected to correct the respiratory mechanics data [2]. Mice were allowed to stabilize on the ventilator for 5 minutes and were then inflated three times to a transrespiratory pressure of 30 cmH_2O to establish a standard volume history,

which corresponds to a recruitment maneuver. FOT was assessed at initiation of mechanical ventilation (before and after volume history standardization), and then repeated hourly to capture the time-course and the detailed response to mechanical ventilation. FOT measurements included a 1.2-second, 2.5 Hz single-frequency signal (SnapShot-150 perturbation) and an eight-second, broadband low-frequency signal containing 17 mutually prime frequencies between 0.5 and 19.75 Hz (Quick Prime-8 perturbation). Respiratory system dynamic resistance was calculated in the FlexiVent© software by fitting the single frequency FOT data to the single compartment model [3].

Histologic examination

An optical microscope (BX51 Olympus) with camera (Camedia 5060, Olympus) was used for histologic examination and photographs. Firstly, all sections were analyzed to evaluate the adequacy of a previously described VILI score used for similar experimental models by other teams [4,5]. This score takes 4 criteria into account: (a) alveolar congestion, (b) hemorrhage, (c) leukocyte infiltration or aggregation of neutrophils in airspace or the vessel wall, and (d) thickness of the alveolar wall, scored on a scale of 0–4. The first three criteria appeared to be unsuitable, as they were either not observed, *i.e.* b) and c) or were nonspecific. The fourth criterion, *i.e.* d) was not sufficiently accurate to describe differences between the observed alveolar lesions. This can be explained by the fact that our model uses protective ventilation instead of high-pressure ventilation. We therefore created a score adapted to the lesions induced by protective ventilation, consisting of specific evaluation of alveolar neutrophil infiltration by semiquantitative scoring.

Flow cytometric analysis

All antibodies, isotypes and clones used are described in Table S1.

Details of the leukocyte identification procedure are represented by the Figure S1.

REFERENCES

1. Allen, G.B., Suratt, B.T., Rinaldi, L., Petty, J.M. & Bates, J.H. Choosing the frequency of deep inflation in mice: balancing recruitment against ventilator-induced lung injury. *Am J Physiol Lung Cell Mol Physiol* **291**, L710-717 (2006).
2. Schuessler, T.F. & Bates, J.H. A computer-controlled research ventilator for small animals: design and evaluation. *IEEE Trans Biomed Eng* **42**, 860-866 (1995).
3. Pillow, J.J., Korfhagen, T.R., Ikegami, M. & Sly, P.D. Overexpression of TGF-alpha increases lung tissue hysteresivity in transgenic mice. *J Appl Physiol (1985)* **91**, 2730-2734 (2001).
4. Belperio, J.A.*, et al.* Critical role for CXCR2 and CXCR2 ligands during the pathogenesis of ventilator-induced lung injury. *J Clin Invest* **110**, 1703-1716 (2002).
5. Imanaka, H.*, et al.* Ventilator-induced lung injury is associated with neutrophil infiltration, macrophage activation, and TGF-beta 1 mRNA upregulation in rat lungs. *Anesth Analg* **92**, 428-436 (2001).

Table S1 : Antibodies, isotypes and clones used for flow cytometry analysis.

Antibodies	Fluorochrome	Isotype	Laboratory	Concentration
Ly6G/Ly6C (Gr-1)	FITC	IgG2a, κ	BD Biosciences, San Jose, CA, USA	2.5 µg / 1 million cells
CD11c	PE	IgG1, λ2	BD Biosciences	1 µg / 1 million cells
F4/80	PE-Cy7	IgG2a, κ	Biolegend	0.25 µg / 1 million cells
CD62L	APC	IgG2a, κ	Biolegend	
CD11b	APC-Cy7	IgG2b, κ	BD Biosciences	

Figure S1

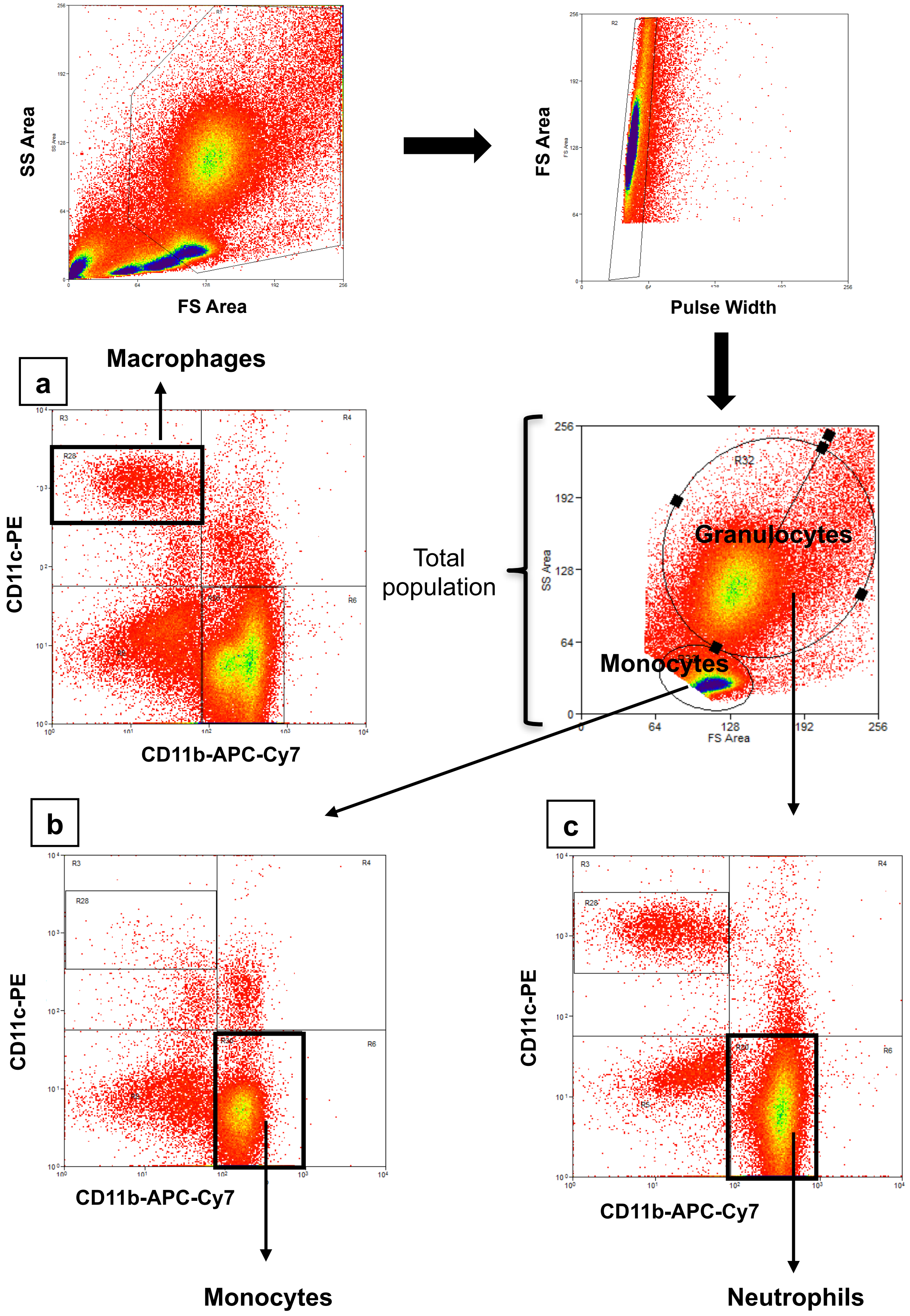
SS Area
FS Area
FS Area
Pulse Width
a
Macrophages
CD11c-PE
CD11b-APC-Cy7
Total population
SS Area
FS Area
Granulocytes
Monocytes
b
CD11c-PE
CD11b-APC-Cy7
Monocytes
c
CD11c-PE
CD11b-APC-Cy7
Neutrophils

FIGURE LEGEND

Figure S1: Figure showing flow cytometry analysis of macrophages, monocytes and neutrophils in mouse lungs.

a. Dot-plot showing CD11b *versus* CD11c expression of the total cell population. Macrophages were identified as high CD11c and low CD11b cells.

b. Dot-plot showing CD11b *versus* CD11c expression of the low side-scatter cell population. Monocytes were identified as low CD11c and high CD11b cells of this low side-scatter population.

c. Dot-plot showing CD11b *versus* CD11c expression of the high side-scatter cell population. Neutrophils were identified as low CD11c and high CD11b cells of this high side-scatter population.
